# Anti-Inflammatory and Anti-Oxidative Effects of AM404 in IL-1β-Stimulated SK-N-SH Neuroblastoma Cells

**DOI:** 10.3389/fphar.2021.789074

**Published:** 2021-11-17

**Authors:** Matthias Apweiler, Jana Streyczek, Soraya Wilke Saliba, Johannes Ditrich, Eduardo Muñoz, Bernd L. Fiebich

**Affiliations:** ^1^ Neuroimmunology and Neurochemistry Research Group, Department of Psychiatry and Psychotherapy, Medical Center-University of Freiburg, Faculty of Medicine, University of Freiburg, Freiburg, Germany; ^2^ Zentrum für Psychiatrie Emmendingen, Emmendingen, Germany; ^3^ Departamento de Biología Celular, Fisiología e Inmunología, Universidad de Córdoba, Córdoba, Spain; ^4^ Instituto Maimónides de Investigación Biomédica de Córdoba, Córdoba, Spain; ^5^ Hospital Universitario Reina Sofía, Córdoba, Spain

**Keywords:** AM404, paracetamol, acetaminophen, prostaglandin E_2_, 8-iso-PGF_2α_, cyclooxygenase, neuroinflammation, oxidative stress

## Abstract

An emerging number of studies address the involvement of neuroinflammation and oxidative stress in the pathophysiology of central nervous system (CNS) disorders such as depression, schizophrenia, anxiety, and neurodegenerative diseases. Different cytokines and molecules, such as prostaglandin (PG) E_2_, are associated with neuroinflammatory processes. The active acetaminophen metabolite AM404 has been shown to prevent inflammation and neuroinflammation in primary microglia and organotypic hippocampal slice cultures. However, its effects on pathophysiological conditions in the CNS and especially on neurons are still poorly understood. In this study, we therefore evaluated the effects of AM404 and acetaminophen on the arachidonic acid cascade and oxidative stress induced by interleukin (IL)-1β in human SK-N-SH neuronal cells. We observed that AM404 and acetaminophen significantly and concentration-dependent inhibited IL-1β-induced release of PGE_2_, independent of cyclooxygenases (COX)-1 and COX-2 enzymatic activity as well as COX-2 mRNA and protein levels in SK-N-SH-cells. The reduction of IL-1β-induced PGE_2_-release by AM404 and acetaminophen treatment might be mediated by the 8-iso-PGF_2α_ pathway since IL-1β-induced synthesis of this free radical marker is dose-dependently reduced by both compounds, respectively. Therefore, understanding of the potential therapeutic properties of AM404 in neuroinflammation and oxidative stress might lead to future treatment options of different neurological disorders.

## Introduction

Neurodegeneration is an important characteristic of different diseases such as Alzheimer’s Disease (AD), Parkinson’s Disease (PD), and other neuropsychiatric diseases (Sultana et al., 2013; Joshi and Praticò, 2014). On the molecular level, different pathomechanisms may induce neurodegeneration such as wrong-folded proteins accumulating in the brain, neuroinflammation, and oxidative stress (Brundin et al., 2010; Rozpędek-Kamińska et al., 2020), with the two latter being closely connected and promoting each other (Picca et al., 2020). Psychiatric disorders, as for example depression, schizophrenia, and anxiety disorders, are also associated with neuroinflammation and oxidative stress (Salim, 2014).

Besides neuroinflammation, oxidative stress is associated with neuronal damage and neuropsychiatric diseases (Sultana et al., 2013; Joshi and Praticò, 2014). An example is the production of 8-iso-prostaglandin (PG) F_2α_ induced by amyloid beta in β-pleated sheet conformations, as part of senile plaques in AD (Mark et al., 2008). Since the CNS is highly metabolic active, reactive oxygen species (ROS) arising of this metabolism need to be cleared to maintain oxidative homeostasis (Singh et al., 2019). Under normal conditions, ROS are neutralized by antioxidants and enzymatic cell processes. If these mechanisms are exhausted, concentrations of ROS rise, damaging parts of the cell, such as proteins, membranes, and lipids, (Joshi and Praticò, 2014). Lipid peroxidation is a consequence of high intracellular ROS-levels resulting in highly reactive aldehydes or cyclized fatty acid esters such as isoprostanes (Sultana et al., 2013). 8-iso-PGF_2α_, also known as 8-isoprostane, is described as sensitive marker for detecting oxidative stress in cells and is associated with cytotoxicity in higher concentrations (Mark et al., 2008). It is formed by lipid peroxidation of arachidonic acid (AA) independent of cyclooxygenases (COXs). In addition to its direct cytotoxic and pro-oxidative effects, 8-iso-PGF_2α_ might potentially act as a signaling molecule by binding to a modified form of isoprostane receptors activating mitogen activated protein kinase (MAPK)-pathways (Acquaviva et al., 2013). Yet, downstream signaling of isoprostanes and possible involvement in the COX-2/PGE_2_ pathway is still poorly understood.

Since acetaminophen, also known as paracetamol, has been introduced to the pharmaceutical market, it has quickly gained importance. It is known to reduce pain and fever, but its molecular mechanisms, besides its effects on prostaglandins, are not fully understood so far. *N*-arachidonoylphenolamine (AM404) is a metabolite of acetaminophen. In the CNS, acetaminophen is deacetylated to phospho-acetaminophen and conjugated with AA by the fatty acid amide hydrolase (FAAH) to form AM404 (Högestätt et al., 2005; Bertolini et al., 2006). Acetaminophen is used as an analgetic and antipyretic drug through inhibition of PG-synthesis but is not suggested to be a potent anti-inflammatory drug (Flower and Vane, 1972). AM404 is discussed as the active metabolite of acetaminophen responsible for its central effects by acting *via* cannabinoid receptor 1 (CB1) or transient receptor potential vanilloid receptor 1 (TRPV1) (Zygmunt et al., 2000; Bertolini et al., 2006). The endocannabinoid system, including CB1 and CB2 receptors and their endogenous as well as exogenous ligands, is an important system and target for the regulation of inflammatory processes (Pacher et al., 2006).

AM404 has been demonstrated to reduce inflammation and oxidative stress. AM404 reduced cluster of differentiation (CD)3/CD28-induced interleukin (IL)-2 release and T-cell proliferation in Jurkat cells, inhibiting NFAT-transcription and transcriptional factor activity (Caballero et al., 2007). The treatment of Sprague Dawley rats with AM404 ameliorated lipopolysaccharide (LPS)-induced IL-1β- and IL-6-levels and increased tumor necrosis factor alpha (TNFα) concentrations in the plasma (Roche et al., 2008). In murine 4-aminopyridine-induced epileptic hippocampal neurons, AM404 reduced anandamide- and capsaicin-induced Ca^2+^-accumulation, apoptosis and ROS-generation (Nazıroğlu et al., 2019). In our previous studies, we showed the reduction of LPS-induced PGE_2_ and 8-isoprostane release by AM404 in concentration-dependent manner in primary rat microglia (Saliba et al., 2017) and N-methyl-D-aspartate (NMDA)-induced IL-1β-expression in organotypic hippocampal slice cultures (OHSC) of mouse brain (Saliba et al., 2019). Furthermore, COX-2 protein levels and COX-activity induced by LPS were significantly reduced by AM404. The observed anti-inflammatory effects were independent of TRPV1, as shown using microglia from TRPV1 knockout mice (Saliba et al., 2017). Microglia are known to be key effectors in neuroinflammation and in inflammatory response to external stressors (Yap et al., 2019). Their response is not only affecting themselves and other immune cells of the CNS, but also triggering inflammatory processes and responses in neurons leading to neurodegenerative, neuropsychiatric, and cognitive symptoms (Najjar et al., 2013; Yap et al., 2019). Neuroinflammation can be described as an imbalance between anti-inflammatory and pro-inflammatory mediators due to internal or external stimuli (Craft et al., 2005). Neuroinflammatory molecules released by microglia and neurons are for instance IL-1β, IL-6, and PGE_2_ (Craft et al., 2005; Lima et al., 2012), the latter is synthesized from AA by enzymatic activity of COX-1/2 and microsomal prostaglandin synthase (mPGES)-1 (Lee et al., 2010).

Neuroprotective effects of AM404 have been also reported *ex vivo* and *in vivo*. In OHSC, AM404 protects against NMDA-induced neurotoxicity (Saliba et al., 2019). In 3xTg-AD mice, systemic low dose treatment with AM404 reduced memory impairment and loss of serotonergic and noradrenergic neurons. Furthermore, serum levels of IL-6 and TNFα were significantly decreased by AM404 treatment. Concentrations used for the treatment of mice were beneath reported minimal concentrations for CB1 and TRPV1 receptor activation. Therefore, the effects shown might be receptor-independent and might be mediated by nonspecific targets, such as anti-oxidative pathways (Huang et al., 2019). Other studies found anxiolytic and antidepressant effects of AM404 in animal models (Bortolato et al., 2006; Patel and Hillard, 2006; Adamczyk et al., 2008) as well as neuroprotective effects in a rat model of PD (Fernandez-Espejo et al., 2004; García-Arencibia et al., 2007). With a more detailed understanding of its molecular mechanisms and effects, AM404 might be an interesting option in the treatment of neurological and psychiatric diseases associated with neuroinflammatory or oxidative processes.

The current study focuses on the effects of AM404 and acetaminophen in human SK-N-SH neuroblastoma cells by evaluating IL-1β-induced neuroinflammatory and oxidative endpoints, such as PGE_2_-and 8-iso-PGF_2α_-concentrations, and the underlying mechanisms.

## Methods

### Chemicals

N-arachidonoylphenolamine (AM404; Alomone Labs, Jerusalem, Israel) was dissolved in DMSO (Merck KGaA, Darmstadt, Germany) and used in final concentrations of 0.1–10 µM. Acetaminophen (APAP; paracetamol; Sigma-Aldrich GmbH, Taufkirchen, Germany) was dissolved in ethanol (Sigma-Aldrich) and used in final concentrations of 1–50 µM. The chosen range of AM404 (Saliba et al., 2017) and acetaminophen (Fiebich et al., 2000) concentrations was based on previous studies. To compare CB1- and CB2-mediated effects on PGE_2_-release, arachidonyl-2′-chloroethylamide (ACEA, CB1 agonist; in ethanol, Biotrend Chemicals AG, Köln, Germany) and (2-Methyl-1-propyl-1H-indol-3-yl)-1-naphthalenylmethanone (JWH-015; CB2 agonist, in DMSO; Tocris Bioscience, Bristol, United Kingdom) were used. Human IL-1β [100,000 U/ml in phosphate buffered saline (PBS)] was purchased from Roche Diagnostics (Manheim, Germany) and used at a final concentration of 10 U/ml in the experiments.

### Human Neuroblastoma (SK-N-SH) Cell Culture

SK-N-SH-cells were obtained from the ATCC (HTB-11, Rockville, United States) and grown in 1× minimum essential medium (MEM) containing Earl’s salts, 10% fetal bovine serum (Bio & SELL GmbH, Feucht/Nürnberg, Germany), 1 mM l-glutamine, 1 mM sodium pyruvate, 2 ml of 100x MEM vitamin solution, 40 units/ml penicillin, 40 μg/ml streptomycin, and 0.1 μg/ml fungizone^®^ (all obtained from Gibco, Thermo Fisher Scientific, Bonn, Germany). Cells were incubated at 37°C in a humidified atmosphere with 5% CO_2_. Confluent monolayers were passaged routinely by trypsinization. After trypsinization, cells were harvested and re-seeded into 6-, 12-, 24-, or 96-well plates. On the next day, medium was changed and after 1 h, cells were stimulated for respective experiments.

### Cell Viability Assay

Viability of SK-N-SH-cells after treatment with AM404 and acetaminophen was measured using MTT assay (Sigma-Aldrich). This assay determines the number of metabolically active and viable cells in cell culture based on the reduction of a yellow tetrazolium salt [3-(4,5-dimethylthiazol-2-yl)-2,5-diphenyltetrazolium bromide or MTT] to purple formazan. Briefly, cells were cultured in 96-well plates at the density of 25 × 10^3^ cells/well for 24 h. Then, medium was changed and after at least 1 h, cells were pre-treated with different concentrations of AM404 or acetaminophen for 30 min. Cells were then incubated with or without IL-1β for the next 20 h. Ethanol (20% end conc.) was used as positive control to induce cell death. Next, 20 µl of MTT-solution (5 mg/ml) were added to all wells and incubated for another 4 h at 37°C. Then, medium was removed and 200 µl of DMSO were added. Colorimetric reaction was measured using MRX^e^ Microplate reader (Dynex Technologies, Denkerdorf, Germany) at 595 nm.

### Determination of PGE_2_-and 8-Iso-PGF_2α_ (8-Isoprostane)-Release

SK-N-SH-cells were pre-treated with AM404 (0.1–10 µM), acetaminophen (0.1–50 µM), ACEA (0.1–10 µM), or JWH-015 (0.1–10 µM) for 30 min. Afterwards, cells were incubated with or without IL-1β (10 U/ml) for the next 24 h and supernatants were collected. The levels of PGE_2_ and 8-iso-PGF_2α_ were measured using commercially available enzyme immunoassay (EIA) kits (Cayman Chemicals, Ann Arbor, Michigan, United States, distributed by BioMol, Hamburg, Germany) following the manufacturer’s protocol. The results were normalized to IL-1β and presented as percentage of change in PG-levels of at least three independent experiments.

### Cyclooxygenase Activity Assay

The COX enzymatic activity was investigated using the AA assay (Fiebich and Chrubasik, 2004). For COX-1 activity, neuroblastoma cells were plated in 24-well plates and after 24 h, medium was removed and replaced with serum-free medium. AM404 (0.1–10 µM) or selective inhibitors of COX-1 [acetylsalicylic acid (ASA, 10 μM), and SC560 (1 μM); Sigma-Aldrich] were added, and left for 15 min. Then, AA (15 μM; Sigma-Aldrich) was applied for another 15 min. Finally, supernatants were collected and used for the determination of PGE_2_.

For COX-2 enzymatic activity, the assay was conducted as described for COX-1, but with pre-incubation of IL-1β (10 U/ml) for 24 h to induce COX-2 synthesis and using diclofenac (1 μM; Sigma-Aldrich) as preferential COX-2 inhibitor.

### Immunoblotting

SK-N-SH-cells were pre-treated with AM404 (0.1–10 µM) for 30 min. After 24 h of IL-1β-stimulation (10 U/ml), cells were washed with cold PBS and lysed mechanically in lysis buffer (42 mM Tris–HCl, 1.3% sodium dodecyl sulfate, 6.5% glycerin, 100 μM sodium orthovanadate, and 2% phosphatase and 0.2% protease inhibitors). Protein concentrations of the samples were measured using the bicinchoninic acid protein assay kit (Thermo Fisher Scientific). For Western blotting, 20 μg of total protein from each sample were subjected to sodium dodecyl sulfate-polyacrylamide gel electrophoresis (SDS-PAGE) under reducing conditions. Proteins were then transferred onto polyvinylidene fluoride membranes (Merck Millipore) by semi-dry blotting. After blocking with Roti-Block (Roth, Karlsruhe, Germany), membranes were incubated overnight with primary antibody [mouse anti-COX-2 (MAB-4198, 1:1000; RD systems, Wiesbaden, Germany). The proteins were detected with horseradish peroxidase-coupled sheep anti-mouse IgG (1:20,000 dilution; Amersham Biosciences GmbH, Freiburg, Germany) using enhanced chemiluminescence (ECL) reagents (Biozym, Hessisch Oldendorf, Germany). Densitometric analysis was performed using ImageJ software (NIH, United States).

### RNA Isolation and Quantitative PCR

For quantification of the mRNA of the enzymes of the COX-2/PGE_2_ pathway, we performed quantitative real-time PCR (qPCR) in SK-N-SH-cells. Cultured cells were pre-treated with AM404 (0.1–10 µM) for 30 min, followed by stimulation with IL-1β (10 U/ml) for 4 h. Total RNA was extracted using the GeneMATRIX Universal RNA Purification Kit (Roboklon GmbH, Berlin, Deutschland), according to the manufacturer’s protocol. Then, cDNA was reverse transcribed from 500 ng of total RNA with initial denaturation at 70°C followed by amplification cycle after addition of master mix. qPCR amplification was carried out by the CFX96 real-time PCR detection system (Bio-Rad Laboratories GmbH, Feldkirchen, Germany). Glyceraldehyde 3-phosphate dehydrogenase (GAPDH) served as an internal control for sample normalization. The primer sequences were GAPDH: Fwd: 5′-TGGGAAGCTGGTCATCAAC-3′/Rev: 5′- GCA​TCA​CCC​CAT​TTG​ATG​TT-3′, COX-2: Fwd: 5′- CTTCACGCATTTCAAG -3′/Rev: 5′- TCACCGTAAAGTCCAC -3′ and mPGES-1: Fwd 5′-TGCAGCACGCTGCTGGTCAT-3′/Rev 5′-GTC​GTT​GCG​GTG​GGC​TCT​GAG-3′. Primers were designed using Universal ProbeLibrary Assay Design Center (Roche Diagnostics) and obtained by biomers.net GmbH (Ulm, Germany).

### ORAC-Assay

The anti-oxidative capacity of AM404 (10 and 25 µM) was evaluated using the OxiSelect™ oxygen radical antioxidant capacity (ORAC) *ex vivo* activity assay (Cell Biolabs, Inc., San Diego, CA, United States) following the manufacturer’s instructions. Briefly, Trolox™ antioxidant standard or test samples were added to a 96-well plate, mixed with fluorescein solution and after 30 min incubation at 37°C, free radical initiator solution was added to all wells. Fluorescence was determined at 37°C using a microplate reader (PerkinElmer Victor X5 2030-0050 Multimode Plate Reader, Rodgau, Germany; excitation wavelength 485 nm, emission wavelength 535 nm). Raw values were transformed to Trolox Equivalents (TE).

### Statistical Analysis

Raw values were converted to percentage and IL-1β (10 U/ml) or the appropriate positive control, such as untreated cells for MTT-assay, were considered as 100%. Data are represented as mean ± SEM of at least three independent experiments. The statistical comparisons were performed using one-way ANOVA with Dunett’s post hoc test (Prism 8 software, GraphPad software Inc., San Diego, CA, United States). The level of significance was set at *p < 0.05, **p < 0.01, ***p < 0.001 and ****p < 0.0001.

## Results

### Effects of AM404 and Acetaminophen on Cell Viability

**FIGURE 1:**
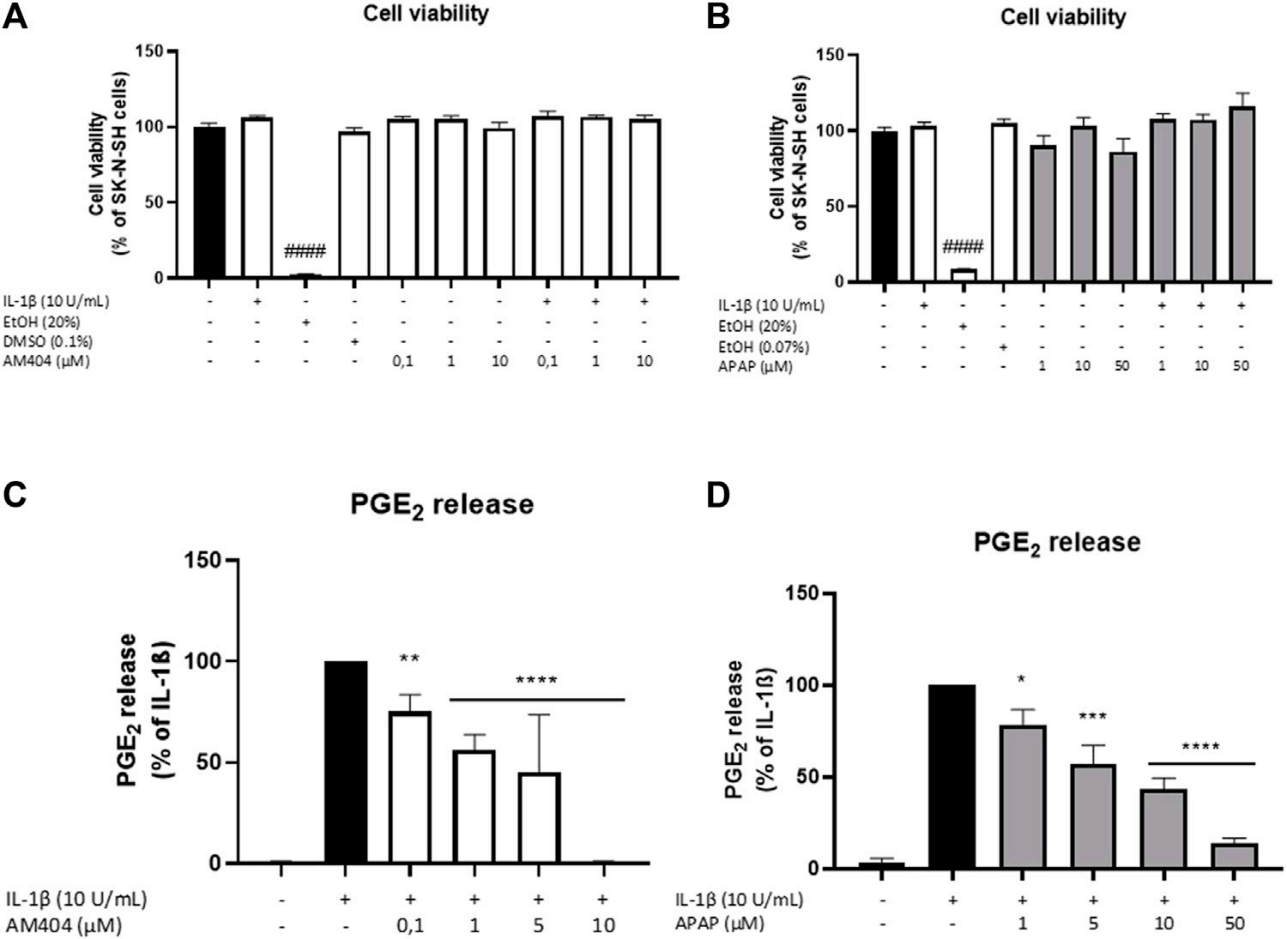
Effects of AM404 **(A,C)** and acetaminophen **(B,D)** on cell viability **(A,B)** and PGE_2_ release **(C,D)** of IL-1β-stimulated SK-N-SH-cells (24 h treatment). Cells were stimulated as described under material and methods. **(A,B)** Cell viability was measured by change in color due to MTT-oxidation and absorbance was measured at 595 nm using an ELISA-reader. **(C,D)** After 24 h of stimulation, supernatants were collected and release of PGE_2_ was measured by EIA. Values are presented as the mean ± SEM of at least three independent experiments. Statistical analysis was performed using one-way ANOVA with Dunett’s post hoc test with **p < 0.01, ***p < 0.001 and ####/****p < 0.0001 compared to untreated cells **(A,B)** or IL-1β **(C,D)**.

We first evaluated the effects of AM404 and acetaminophen on cell viability of SK-N-SH-cells. As shown in Figure 1, neither AM404 (Figure 1A) nor acetaminophen (Figure 1B), significantly reduced cell viability in the used concentrations with or without IL-1β-treatment compared to unstimulated cells, whereas 20% ethanol significantly induced cell death. DMSO and ethanol as solvents of AM404 and acetaminophen, respectively, did not affect cell viability in the concentrations used for the experiments.

### Effects of AM404 and Acetaminophen on IL-1β-Induced PGE_2_-Release

We next evaluated the effects of non-toxic doses of AM404 and acetaminophen on IL-1β-induced PGE_2_-release in human SK-N-SH neuroblastoma cells. As shown in Figure 1C, IL-1β potently induced an increase of PGE_2_-release compared to the untreated control and this effect was, in a concentration-dependent manner, significantly inhibited by all concentrations of AM404 (0.1–10 µM). Acetaminophen (1–50 µM) also showed significant and concentration-dependent inhibitory effects on IL-1β-stimulated PGE_2_-release (Figure 1D).

ACEA, a CB1 agonist, significantly increased IL-1β-induced PGE_2_-release, whereas JWH-015, a CB2 agonist, weakly reduced IL-1β-mediated PGE_2_-synthesis by approximately maximal 50% in SK-N-SH-cells (Supplementary Figure S1). Both CB receptors are expressed in SK-N-SH-cells (data not shown).

### Effects of AM404 on COX-1 and -2 Expression and COX-2 Protein Levels

**FIGURE 2:**
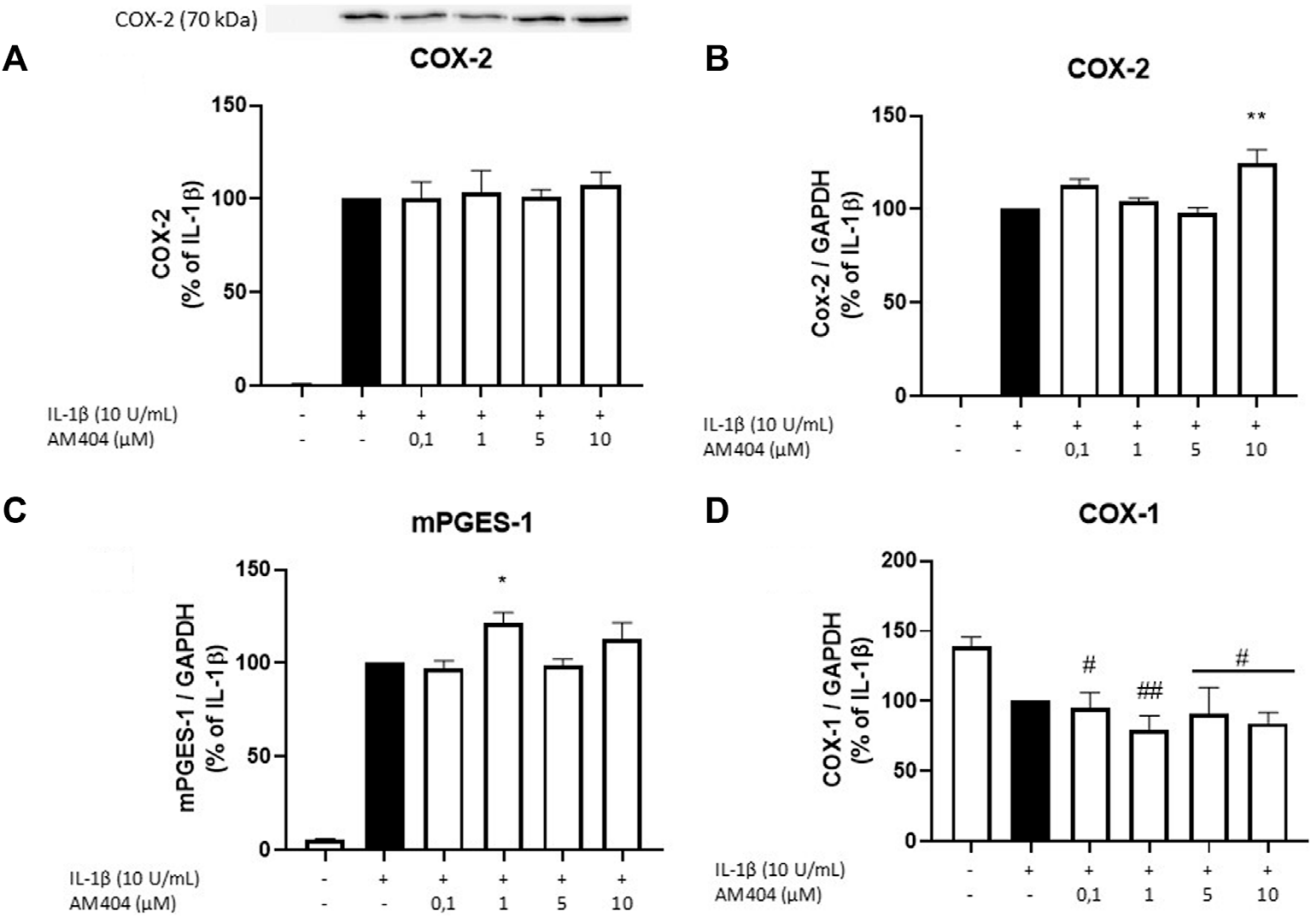
Effects of AM404 on COX-2 protein levels **(A)**, COX-2 expression **(B)**, mPGES-1 expression **(C)**, and COX-1 expression **(D)** in IL-1β-stimulated SK-N-SH-cells. Cells were stimulated as described under material and methods. After 4 h of stimulation, RNA was isolated and mRNA levels of the shown target genes were measured in qPCR. Values are presented as the mean ± SEM of at least three independent experiments. Statistical analysis was performed using one-way ANOVA with Dunnett’s post hoc tests with */#*p* < 0.05, **/##*p* < 0.01 compared to IL-1β **(A–C)** or to untreated cells **(D)**.

There are two known COX enzymes; COX-1 is constitutively expressed in most tissues, whereas COX-2 is mainly induced by inflammatory stimuli but also constitutively expressed in some cells (Samuelsson et al., 1975; Sigal, 1991). Most PGs are synthesized during inflammation by COX-2 and mPGES-1 enzymes (Kudo and Murakami, 2005). We evaluated effects of AM404 on COX-2 protein levels and COX-1, COX-2, and mPGES-1 mRNA expression. As shown in Figure 2, IL-1β potently induced COX-2 protein synthesis and COX-2 and mPGES-1 mRNA expression. COX-2 protein levels were not affected by AM404 treatment (Figure 2A), while mRNA-expression was slightly but still significantly enhanced in concentrations of 10 µM (Figure 2B). The expression of mPGES-1 was marginally but still significantly increased in the concentrations of 1 µM AM404 (Figure 2C). COX-1 mRNA-expression (Figure 2D) was decreased by IL-1β compared to untreated cells. AM404 did not reverse IL-1β-induced reduction of COX-1 mRNA.

### Effects of AM404 on COX Activity

**FIGURE 3:**
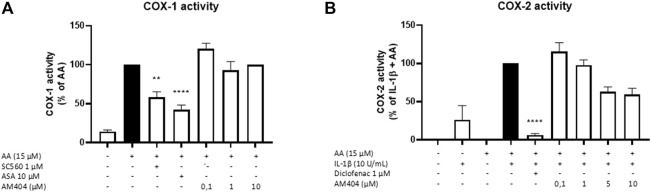
Effects of AM404 on COX-1 enzyme activity **(A)** and COX-2 enzyme activity **(B)** in SK-N-SH-cells. **(A)** COX-1-activity was measured after 15 min of incubation with arachidonic acid (AA). Levels of PGE_2_ in the supernatants were quantified by EIA. **(B)** After 24 h IL-1β-pre-stimulation, 15 µM of AA was added and PGE_2_-release was measured by EIA. Values are presented as the mean ± SEM of at least three independent experiments. Statistical analysis was carried out by using one-way ANOVA with Dunnett’s post hoc tests with **p < 0.01, ****p < 0.0001 compared to AA **(A)** or IL-1β with AA **(B)**.

Since PGE_2_-levels induced by IL-1β are inhibited by AM404 treatment, we investigated whether AM404 directly affected COX enzymatic activity as shown for most non-steroidal anti-inflammatory drugs such as acetyl salicylic acid (ASA), diclofenac, ibuoprofen, and many others. We observed that AM404 did not affect COX-1 activity (Figure 3A) and partially but not significantly (±40%) inhibited COX-2 activity in the doses of 5 and 10 µM (Figure 3B). Known inhibitors of COX-1 (SC560, ASA) and COX-2, (diclofenac) showed a prominent reduction of COX-1 or COX-2 activity. Acetaminophen does not affect COX-activities as shown in multiple studies before (Ohashi and Kohno, 2020).

### Effects of AM404 on Oxidative Stress and as Anti-Oxidative Molecule

**FIGURE 4:**
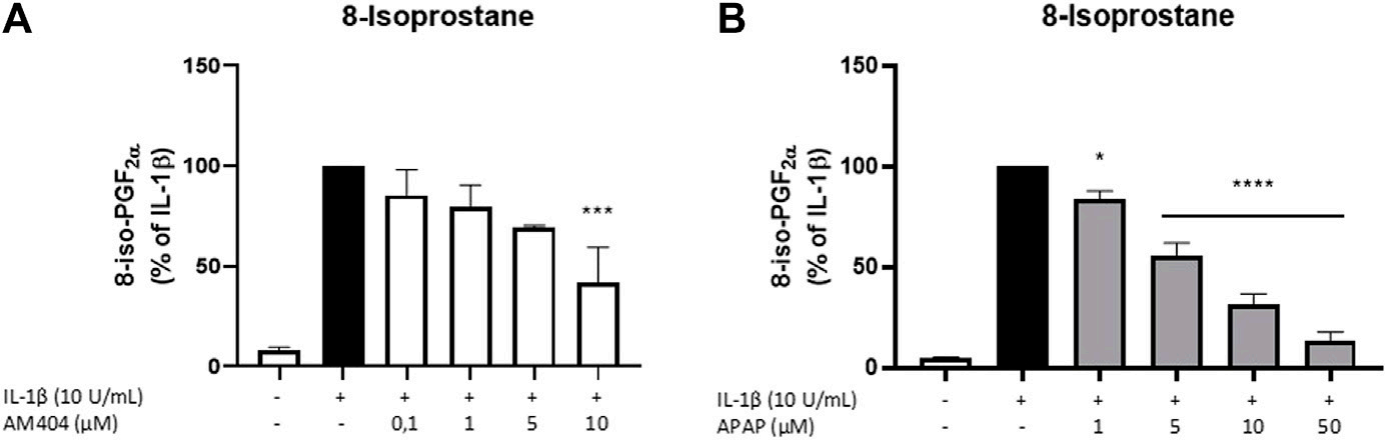
Effects of AM404 **(A)** and acetaminophen **(B)** on 8-iso-PGF_2α_-release in IL-1β-stimulated SK-N-SH-cells. Cells were stimulated as described under material and methods. After 24 h of stimulation, supernatants were collected and release of 8-iso-PGF_2α_ was measured by EIA. Values are presented as the mean ± SEM of at least three independent experiments. Statistical analysis was performed using one-way ANOVA with Dunnett’s post hoc tests with **p < 0.01, ***p < 0.001, ****p < 0.0001 compared to IL-1β.

Since the reduction of IL-1β-induced PGE_2_-levels after treatment with AM404 cannot be explained by changes in COX-expression nor enzyme synthesis or activity, we investigated anti-oxidative mechanisms underlying the PGE_2_-reduction. Therefore, we evaluated the effects of AM404 and acetaminophen on IL-1β-induced 8-Iso-PGF_2α_-release in SK-N-SH-cells. The effects of AM404 and acetaminophen on IL-1β-induced 8-iso-PGF_2α_-release are shown in Figure 4. IL-1β stimulation strongly induced the release of 8-iso-PGF_2α_ compared to untreated cells. AM404 (Figure 4A) as well as acetaminophen (Figure 4B) reduced IL-1β-mediated 8-iso-PGF_2α_-release in a concentration dependent manner. A significant reduction of IL-1β-induced 8-iso-PGF_2α_ was observed in the concentrations of 10 µM of AM404 and starting with 1 µM of acetaminophen to levels close to untreated cells using the dose of 50 µM.

The anti-oxidative capacity of AM404 was determined in comparison to Trolox. AM404 showed around half of the anti-oxidative capacity of the vitamin E analog Trolox, with 6.8 µM of Trolox being as effective as 10 µM AM404 and 13.2 µM of Trolox being as effective as 25 µM AM404.

## Discussion

The current study demonstrates that acetaminophen and its active metabolite AM404 significantly and concentration-dependently reduced the release of IL-1β-induced PGE_2_. The PGE_2_ inhibiting effects of AM404 are independent of COX-1 and COX-2 enzymatic activity. In addition, the observed AM404 effects on PGE_2_-levels are also independent of COX-2 protein as well as COX-2 and mPGES-1 mRNA levels. We demonstrate here that IL-1β-induced 8-iso-PGF_2α_-release [a reliable and highly sensitive marker to assess oxidative stress (Mark et al., 2008)] was significantly decreased by acetaminophen and AM404, and therefore might participate in the decrease of PGE_2_-synthesis.

As shown in our previous study (Saliba et al., 2017), AM404 prevented the synthesis of PGE_2_ and 8-iso-PGF_2α_ in LPS-stimulated primary rat microglia independent of its suggested target receptors CB1 and TRPV1. In primary rat microglia, we found slightly decreased COX-2 and no effect on mPGES-1 protein levels. Furthermore, AM404 reduced COX-1 and COX-2 enzymatic activity, contributing to the observed inhibitory effects on the prostaglandins.

Activated microglia are understood as inflammation driving cells in the CNS, with neurons contributing to inflammation in smaller ways but being especially affected by inflammatory processes (Yap et al., 2019). Therefore, we evaluated the role of AM404 on the neuroinflammatory COX-2/PGE_2_ pathway in human SK-N-SH neuroblastoma cells to replenish the previous microglial results with neuronal cell experiments. In contrast to our microglial results, AM404 reduced IL-1β-induced PGE_2_-and 8-iso-PGF_2α_-release in SK-N-SH-cells, independent of COX enzymatic activity, protein, and mRNA levels.

Oxidative stress is closely connected to neuroinflammation with both conditions promoting each other (Solleiro-Villavicencio and Rivas-Arancibia, 2018; Picca et al., 2020), and the modulation of oxidative stress by anti-oxidative compounds has been reported to decrease neuroinflammation (Simpson and Oliver, 2020). Therefore, inhibition of ROS-generation might also reduce proinflammatory parameters such as PGE_2_. Since 8-iso-PGF_2α_-synthesis is independent of COX enzymatic activity and protein levels, AM404 might reduce IL-1β-induced PGE_2_-levels without affecting COX-2 enzyme activity, protein, or mRNA levels (Watkins et al., 1999). Therefore, PGE_2_-levels might be decreased AM404-dependently *via* ROS and 8-iso-PGF_2α_ pathways (Ting and Khasawneh, 2010; Acquaviva et al., 2013). However, the role of COX in the production of 8-iso-PGF_2α_ is discussed controversially, with some authors suggesting an COX enzyme-dependent generation of isoprostanes (Bauer et al., 2014). Nevertheless, COX-2 activity is associated with much higher concentrations of PGF_2α_ instead of 8-iso-PGF_2α_ (van ’t Erve et al., 2015), thus, regulation of 8-iso-PGF_2α_-levels seems to be COX-2 independent. Further research is necessary to fully understand the role of 8-iso-PGF_2α_ as potentially signaling molecule in the COX-2/PGE_2_ pathway.

Since the role of COX-2 remains discussed controversially (Kopschina Feltes et al., 2017) and may not only exert pro-inflammatory but neuroprotective effects in special constellations as well, the lack of effects of acetaminophen and its metabolite AM404 on COX enzymatic activity and protein levels might be beneficial for therapeutic use. The expression of mPGES-1, the enzyme converting PGH_2_ to PGE_2_, is reliably induced by IL-1β in SK-N-SH-cells. As shown for microglia (Saliba et al., 2017), AM404 does not show any concentration-dependent effects on mPGES-1 expression in SK-N-SH-cells, although it shows a significant increase of mPGES-1 mRNA levels at concentrations of 1 µM. Unfortunately, SK-N-SH cells show high levels of basal mPGES-1 immunoreactivity with only minor induction by IL-1β. This might be due to non-specific binding of the antibody used to other constitutive PGES isoforms such as cPGES or mPGES-2. Hence, this important final step of the COX-2/PGE_2_ pathway cannot be examined on the protein level in SK-N-SH-cells.

Another possible mechanism to explain the decrease of PGE_2_-and 8-iso-PGF_2α_-release might be through intracellular calcium concentrations. Since AA, a necessary substrate in the synthesis of PGE_2_ and 8-iso-PGF_2α_, is mobilized calcium-dependent by cytosolic phospholipase A_2_ (Leslie, 2015), decreasing intracellular calcium concentrations may lead to reduced PGE_2_-and 8-iso-PGF_2α_-synthesis due to lack of substrate. We and others have demonstrated that AM404 decreased intracellular calcium responses (Alptekin et al., 2010; Kerckhove et al., 2014; Saliba et al., 2019) and therefore AM404 might reduce in consequence of decreased intracellular calcium levels the mobilization of AA as substrate for PGE_2_-and 8-iso-PGF_2α_-synthesis.

One other possible mechanism how AM404 is affecting PGE_2_-levels might be an interaction with the peroxidation site of COX-2, which has also been proposed for acetaminophen and ascorbic acid (Fiebich et al., 2000; Boutaud et al., 2002; Candelario-Jalil et al., 2006).

AM404 is suggested to enfold its central effects *via* CB1 or TRPV1 (Zygmunt et al., 2000; Bertolini et al., 2006). Our results, however, suggest a CB1- and TRPV-1 independent decrease of PGE_2_-levels in SK-N-SH-cells, since CB1 receptor agonists, such as ACEA, are associated with increased PGE_2_-concentrations as also confirmed by others (Mitchell, 1993). CB2 agonists, such as JWH-015, indeed are reducing PGE_2_-release (Zoppi et al., 2014), but we found that JWH-015 only reduced IL-1β-induced PGE_2_-levels maximal 50% in the high dose of 10 µM. Therefore, CB2 agonism does not explain the strong reduction of PGE_2_-levels by AM404. Furthermore, the concentrations of AM404 necessary to bind and activate CB2 receptors are much higher compared to ACEA or JWH-015 (CB1: Ki ACEA 1.4 nM; Ki JWH-015 383 nM; Ki AM404 1.5 μM; CB2: Ki ACEA 3.1 μM; Ki JWH-105 13.8 nM; Ki AM404 1.3 µM) (An et al., 2020). In primary mouse microglia isolated from TRPV-1 knockout mice, the decreasing effects of AM404 on PGE_2_ are not affected suggesting also in SK-N-SH-cells an TRPV-1 independent mechanism (Saliba et al., 2017). Therefore, we conclude that the observed PGE_2_ inhibiting effects of AM404 are not mediated by CB1 and TRPV1. A partially effect of CB2 agonism is not very likely but cannot be excluded.

As neuroinflammatory and oxidative processes may extensively be involved in neurological and psychiatric diseases such as PD and AD, and depression (Bouayed et al., 2009; Najjar et al., 2013), targeting these processes opens new therapeutical approaches to these diseases. Clinical studies indicate that pro-inflammatory markers, such as PGE_2_, are frequently and prolonged elevated in patients with major depression (Miller et al., 2009). The depressive-like behavior occurring in rats after overexpression of the COX-2/PGE_2_ pathway in hippocampi and being reversed by COX-2 inhibition underlines the hypothesized involvement of PGE_2_ in neuropsychiatric diseases (Song et al., 2018).

Biomarkers for lipid peroxidation such as 8-iso-PGF_2α_ are found in higher concentrations in psychiatric (Joshi and Praticò, 2014) and neurodegenerative diseases (Sultana et al., 2013). The shown reduction of IL-1β-induced 8-iso-PGF_2α_-levels due to AM404 and acetaminophen pretreatment of SK-N-SH-cells might therefore be beneficial in the therapy of oxidative stress related neuropsychiatric diseases. Furthermore, AM404 shows half of Trolox’s anti-oxidative capacity in the ORAC-assay, underlining momentous anti-oxidative properties of AM404. Therefore, future research elucidating the role of 8-iso-PGF_2α_ in inflammatory signaling processes as well as studying the effects of AM404 in animal models of psychiatric or neurodegenerative disorders might revolutionize future therapies. As immune therapies gain more relevance in the treatment of neuropsychiatric diseases (Najjar et al., 2013), acetaminophen might be an alternative as well-established drug with only mild side effects compared to corticosteroids or immunosuppressive drugs.

However, the connection between neuroinflammation and oxidative stress is complex and therefore a separated view of both components might be to simplifying. Understanding the interactions between the involved players and the initiator of clinical observed symptoms might lead to an individualization of treatment by using biomarkers of neuroinflammation and oxidative stress to choose and monitor therapies of neurological and psychiatric diseases, for instance.

## Conclusion

Many studies have been performed to elucidate the mechanisms of action of acetaminophen and to better understand its effects in different cell types. We show here, that the active acetaminophen metabolite AM404 as well as acetaminophen show significant anti-neuroinflammatory effects by potently inhibiting IL-1β-induced PGE_2_-release in human SK-N-SH-cells. These effects might be mediated by 8-iso-PGF_2α_-reduction as observed for both, AM404 and acetaminophen. Further understanding of the role of AM404 and acetaminophen in neuroinflammation might revolutionize the use of acetaminophen besides its common application in pain and fever and might lead to future treatment of different psychiatric and neurological disorders with a neuroinflammatory background.

## Data Availability

The raw data supporting the conclusion of this article will be made available by the authors, without undue reservation.
