# Supplementary figures and images for: Anti-Inflammatory and Anti-Oxidative Effects of AM404 in IL-1β-Stimulated SK-N-SH Neuroblastoma Cells
Source: Front Pharmacol. 2021 Nov 17;12:789074. doi: 10.3389/fphar.2021.789074 (PMC8635764; doi:10.3389/fphar.2021.789074)

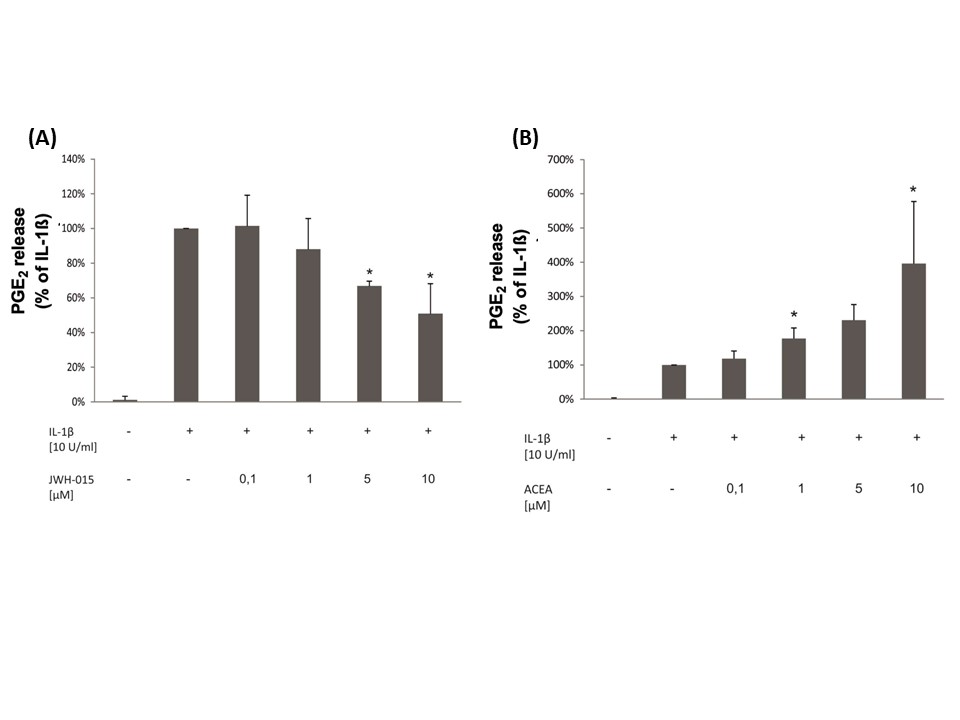

Supplement: Supplementary file 1 [file Image1.JPEG]
