# Supplementary material for: Anti-Inflammatory and Anti-Oxidative Effects of AM404 in IL-1β-Stimulated SK-N-SH Neuroblastoma Cells
Source: Front Pharmacol. 2021 Nov 17;12:789074. doi: 10.3389/fphar.2021.789074 (PMC8635764; doi:10.3389/fphar.2021.789074)
Supplement: Supplementary file 2 [file Image2.pdf]

Western Blot COX-2 (70 kDa) in SK-N-SH-cells (Antibody: R&D Systems, MAB-4198; 1:1000)

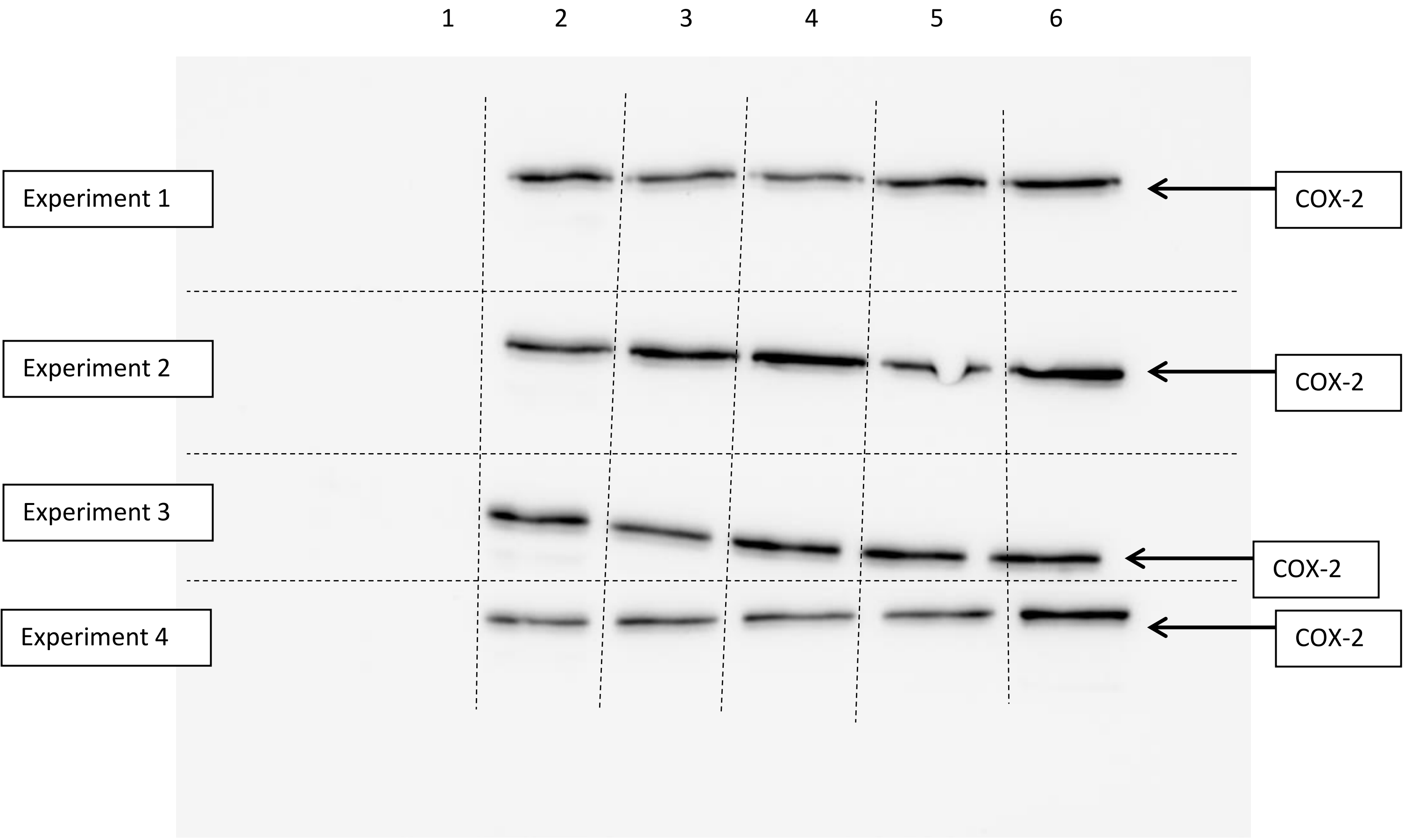

1= Negative Control

2= IL-1β (10 U/mL)

3= 0.1 µM AM404 + IL-1β (10 U/mL)

4= 1 µM AM404 + IL-1β (10 U/mL)

5= 5 µM AM404 + IL-1β (10 U/mL)

6= 10 µM AM404 + IL-1β (10 U/mL)
